# Artery Pulse Waveform Acquired with a Fabry-Perot Interferometer

**DOI:** 10.3390/s24092855

**Published:** 2024-04-30

**Authors:** Sergio Calixto, Zacarias Malacara-Hernandez, Guillermo Garnica, Ingrid Chavez-Serrano

**Affiliations:** Centro de Investigaciones en Optica, Loma del Bosque 115, León 37150, Mexico; zmalacar@cio.mx (Z.M.-H.); garnica@cio.mx (G.G.); ingridcs@cio.mx (I.C.-S.)

**Keywords:** pulsometer, artery pulse waveform, Fabry-Perot interferometer

## Abstract

For most patients admitted to a hospital, it is a requirement to continuously monitor their vital signs. Among these are the waveforms from ECG and the pulmonary arterial pulse. At present, there are several electronic devices that can measure the arterial pulse waveform. However, they can be affected by electromagnetic wave radiation, and the fabrication of electronic sensors is complicated and contributes to the e-waste, among other problems. In this paper, we propose an optical method to measure arterial pulse based on a Fabry-Perot interferometer composed of two mirrors. A pulse sensor formed by an acrylic cell with a thin membrane is used to gather the vasodilatation of the wrist, forming an air pulse that is enacted by means of a tube to a metallic cell containing a mirror that is glued to a thin silicone membrane. When the air pulse arrives, a displacement of the mirror takes place and produces a shift of the interference pattern fringes given by the Fabry-Perot. A detector samples the fringe intensity. With this method, an arterial pulse waveform is obtained. We characterize this optical device as a test of concept, and its application to measuring artery pulse is presented. The optical device is compared to other electronic devices.

## 1. Introduction

Optical sensors play an increasingly important role in the development of medical diagnosis, as described by Kaur et al. [1]. Since olden times, arterial pulse pressure diagnosis has been a principal method of medical practice in China [2]. The doctor’s hands touched parts of the body, and a diagnosis was obtained. The Greek physician Claudius Galen Nicon of Pérgamon (born in the year 169, who wrote the book “De Pulsibus”) already knew the Chinese practice. Around 1682, Michael Boym translated Chinese medicine books into Latin, and, in this way, Chinese knowledge was introduced to Europe. Later (1700), Sir John Floyer studied the Chinese writings and, combined with the Galenic medicine books and with the help of watchmaker Samuel Watson, developed the first pulse watch. They introduced the method of pulse rate measurement [3].

At present, a “pulse oximeter” is used to roughly measure human arterial pulse rate [4]. This instrument measures human oxygen saturation (SpO2), pulse rate value, and the waveform of the pulse. It works on the difference caused by the light absorption change in oxyhemoglobin [5]. However, the ability to measure small changes in the arterial pulse pressure waveform using devices attached to the skin [6] provides some useful physiological characteristics. From this, doctors can evaluate a patient’s vital signs, such as stiffness of arteries, to diagnose hypertension, corona atherosclerosis, and other cardiovascular diseases. Nowadays, these techniques are used in clinical settings. These waveforms are complementary to the electrocardiogram (ECG) and EEG studies.

At present, there are instruments that can measure the arterial pulse pressure waveforms in detail, but they are devices based on electronic technology. For example, a capacitive pressure sensor, which makes wrist pulse measurements, is described in [7]. A two-layer sensor is based on a flexible capacitive pressure device with hierarchically micro-structured electrodes that show high sensitivity. The material used in the top layer is a biaxially oriented polypropylene (BOPP) film sputtered with platinum (Pt) on its top, serving as a dielectric layer. The bottom layer is a pyramidally micro-structured PDMS film, also sputtered with a platinum layer (20–30 nm thick). An impedance analyzer is used to measure the capacitance. Values of 27 pf at 0 kPa and 197 pf at 100 kPa were found.

In addition to the example above, electronic, on-skin flexible radial artery pulse pressure measurement is proposed in [8]. The sensor consists of MXene (Ti3C2Tx)-coated nonwoven fibers (n-WFs) with a pressure-sensitive layer and laser-engraved interdigitated copper electrodes. It shows the conductivity paths between the fibers and electrodes during compression, with a sensitivity of 3.18 kPa-1, a response time of 15 ms, and a low detection pressure of 11 Pa. A wirelessly flexible processor circuit was connected to the sensor, and the ensemble was mounted on the wrist radial artery, sending a signal to a smartphone. The MXene and n-WFs come into contact with each other when the sensor is squeezed by a weak pulse, thereby reducing the contact resistance between the fibers.

Another electronic device [9] made from an inorganic piezoelectric material and a semiconductor (SiNM, n-MOSFET), with both mounted on a thin elastomer substrate (Silicone), was formed as a sensor. The semiconductor enabled the device to connect with the T-Mobile network. The inorganic piezoelectric material was lead zirconate titanate (PbZrTiO3, PZT). The structure of the sensor consisted of an array of lead zirconate titanate squares (560 μm × 560 μm), forming an 8 × 8 array laid on silicone. The squares were collectively connected to a SiNM n-MOSFET. An anisotropic conductive film placed over the silicone was used to connect external power supplies and measurement devices. Blood pressure wave measurements were made, and a response time of 0.1 ms was found. Ref. [10] provides another study similar to this.

Recently, a material that has proven effective at measuring the arterial waveform is the poly(3,4-ethylene doixythiophene): polystyrene sulfonate (PEDOT:PSS) [11,12]. The physical basis of this material is the flexoelectric effect. It is an alternative transduction mechanism to piezoelectricity. PEDOT:PSS films work based on electrical energy generation induced by dynamic bending, and it is used to monitor human artery pulse.

In 1996, a study comparing the aortic and carotid pulse waveforms was presented. The carotid pulse was taken using the tonometry technique, and the aortic pulse was taken by inserting a micro-manometer-tipped catheter into the ascending aorta of 62 adult patients. The tonometer test gave better results [13].

Lastly, an optical pulse wave measurement device based on optical fibers was studied recently [14,15]. The structure of the miniature Fabry-Perot configuration consisted of two perpendicularly cleaved optical fiber end-faces inserted into a glass capillary. The light was guided through an SMF-28 fiber, which then returned the light reflected by the Fabry-Perot cavity to a data board. Together with the end of the second fiber, they were inserted and glued in a capillary with an inner diameter of 130 µm and an outer diameter of 350 µm. The length of the capillary was 1 cm. The ends of the two fibers were 56 µm apart. The light source was an Exalos EXS210066-01 SLED with a spectrum interval between 1.51 µm to 1.59 μm. Two miniature Fabry-Perot interferometers were used: one placed in the wrist and the other in the carotid artery. The raw signals of these two interferometers were sent to a computer, and there, a fast Fourier transform was made. The instrumentation needed was a SLED Exalos EBD5000 driver board, an Ibsen I-MON USB512 spectrometer, an optical fiber circulator, and a computer. There were some slow parasitic components in the raw signals due to the movement of the patient and other factors, and these were removed with a band-pass filter to obtain the pulse waveform. It should be noted that the ends of the optical fibers had a reflection of 4%; thus, the interference pattern consisted of an almost sinusoidal signal close to that of a Michelson interferometer (rather than a Fabry-Perot interferometer) with mirrors, presenting a high reflectance. This interference pattern presents very sharp peaks, which increases its sensitivity.

The electronic sensors described previously present good characteristics, but, unfortunately, electromagnetic radiation could interact with them, thus changing their behavior. In addition, the fabrication of electronic sensors is complicated and requires special materials, dedicated personnel, and expensive fabrication machines; the whole process is long, giving rise to possible problems, and it is not ecological. These sensors contribute highly to e-waste. On the other hand, optical sensors are immune to electromagnetic interference, present high sensitivity, have low cost, are corrosion resistant, are usable in harsh environments, and are non-invasive in a patient’s body, just to mention a few of their good characteristics.

Here, we introduce an optical method based on a Fabry-Perot interferometer [16,17] to measure arterial pulse pressure waveforms. This instrument is composed of two mirrors that present high reflectance. One mirror remains fixed while the other attaches to a thin elastic silicone membrane, which, in turn, is fixed to an extreme of an aluminum circular cell. On the other side of the cell, a flat glass closes the cavity hermetically. The cell is connected to the pulse sensor with plastic tubes. This sensor consists of a circular acrylic cell that has a thin silicone membrane on one side and is closed with flat glass on the other side. On the cell rim, a tube connects to the Fabry-Perot component. The sensor membrane is placed in contact with the artery of the wrist or over the carotid artery. The sensor membrane stretches when a weak blood pulse passes through the artery, creating an air pulse that will travel through the tubing and will reach the Fabry-Perot cell containing the mirror, which then displaces axially. This movement affects the circular fringes of the interference pattern, which shows radial displacement that is recorded by a detector.

Section 2 describes the principles of this interferometer. Section 3 presents the silicone characteristics. Section 4 describes the theoretical arterial pulse waveform, and Section 5 presents the experimental optical configuration and the arterial pulse waveform results. In Section 6, our conclusions are presented.

## 2. The Interferometer Principle

**Figure 1:**
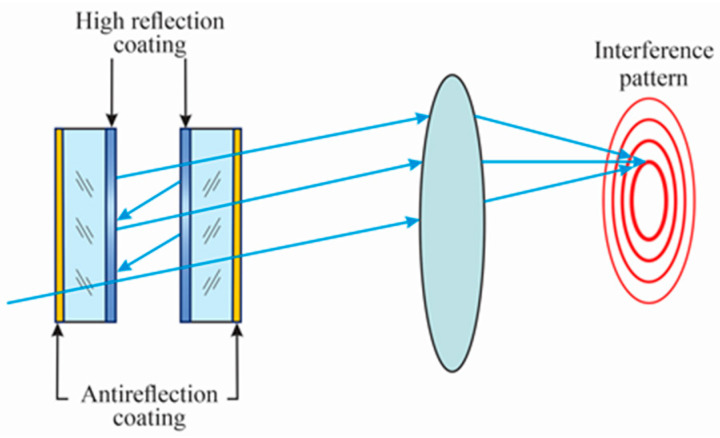
Scheme of a Fabry-Perot interferometer. The set of rings is the interference pattern.

A Fabry-Perot interferometer consists of two high-reflectance mirrors that face each other (Figure 1). Light crosses the first mirror and is reflected many times in the cavity between the mirrors. At the output of the interferometer, a set of light rings is present. The mirrors have a thin film or a stack of thin films with high reflectance on the inner surfaces. This high reflectance affects the width of the light rings.

Equation (17) describes the FP interference pattern:

$$I_{t}=\frac{I_{0}}{\left(1+\left(\frac{4r^{2}}{{\left(1-r^{2}\right)}^{2}}\right){sin}^{2}\left(\frac{\delta }{2}\right)\right)}$$

Here, *δ* = (4π/λ) ns cos ϕ, s is the spacing between the inner faces of the mirrors, n is the refractive index of the material between the mirrors, *r*^2^ is the reflectance of the mirrors, and λ is the wavelength (λ = 632.8 nm for a He-Ne laser).

## 3. Silicone Characteristics

For the precise contact and accurate conversion of the local deformation of the membrane sensor (resulting from the expansion/contraction of the artery) into electrical signals, we chose silicone as the membrane material. This material has proven to be advantageous for creating thin membranes due to its malleability compared to other polymeric materials. The material selected for this project was the high-strength translucent silicone rubber composite known as SILASTIC T-2 [18]. This mixture is composed of a Silastic^®^ T-2 curing agent and a Silastic^®^ T-2 translucent base, which can cure at standard room temperature.

The silicone consists of a siloxane unit (Si-O-Si) used as an elastomer. It is widely used due to its exceptional weather resistance, wide temperature resistance range, and excellent electrical properties. The use of polysiloxanes is particularly widespread in commercial applications. Polysiloxanes have a wide range of practical uses, such as mold-forming, water-repellent molds, anti-foaming agents, lubricants, seals, gaskets, masonry additives, hydraulic fluids, and pressure-sensitive adhesives. These substances exhibit marked physical characteristics, such as a low glass transition temperature (Tg), low viscosity, a low thermal coefficient of viscosity, high gas permeability, low surface energy, and low surface tension, according to sources [19,20].

The specific properties of a mixture consisting of 100 parts translucent base and 10 parts curing agent include a viscosity of 550 cp/mPa.s, a transparent color, a specific gravity of 1.2, a hardness of 42 points, elongation up to 300%, and stress tension of 800 psi. For an in-depth preparation of the samples and their mechanical characteristics, see [21].

## 4. Arterial Pulse Pressure Waveforms

**Figure 2:**
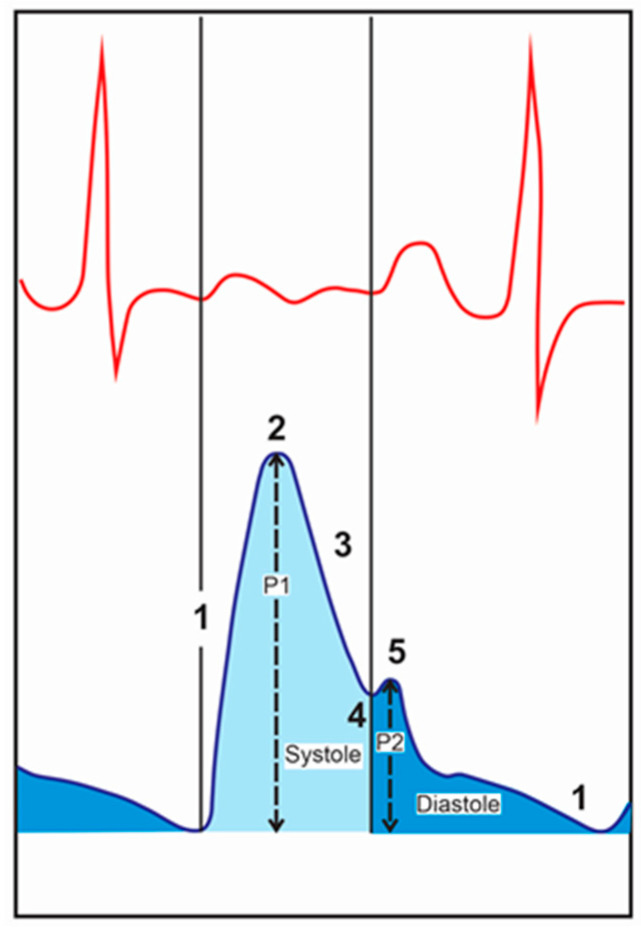
Electro-cardiogram pulse and arterial pulse waveforms [6].

Figure 2 shows both the basic arterial pulse pressure waveform together with an electrocardiogram pulse, as described in [6]. The plot section from point 1 to point 2 of the waveform occurs due to two phenomena: (a) the ventricle ejects the blood into the aorta, and (b) the wave from the narrower vessels reflected back is recorded. At point 2, the maximum volume displacement is present. In Section 3, the ventricles empty, and the blood flow happens slowly. Then, the aortic valve closes, producing the dicrotic valley at point 4. At point 5, the pulmonic valve closes, and atrioventricular valves open. The distance from the base to point 2 is called P1, and the distance from the base to point 5 is called P2. The ratio P2:P1 is the radial artery augmentation index (AIr), and the time difference between the two peaks, ΔTDVP, is the time difference between the peaks and represents the measure of arterial stiffness.

## 5. The Experimental Optical Configuration

### 5.1. The Experimental Fabry-Perot System

**Figure 3:**
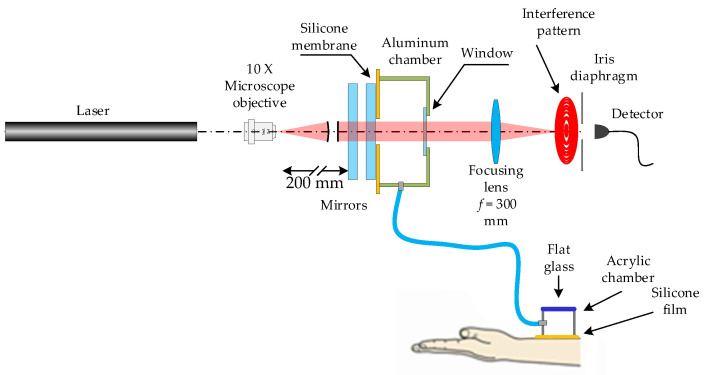
Fabry-Perot interferometer configuration to measure blood pressure.

**Figure 4:**
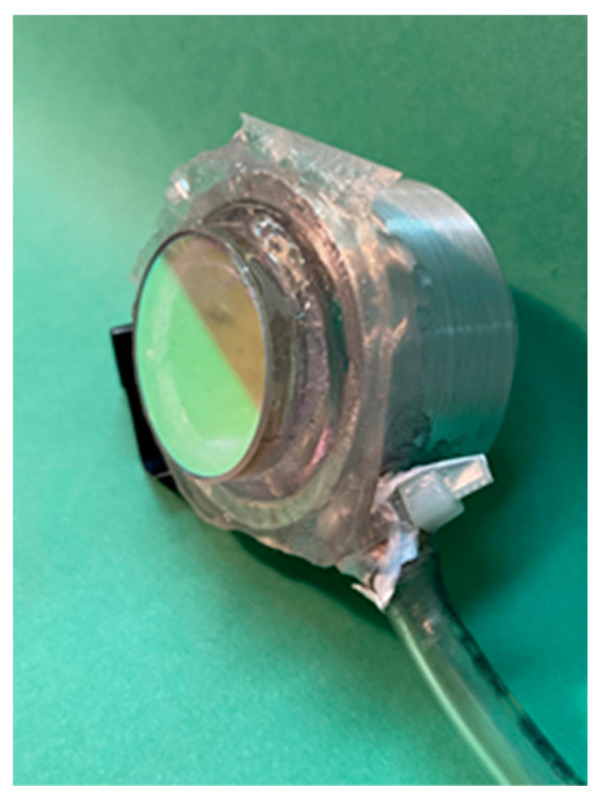
Metallic cell. On one side, it has a thin, flexible silicone membrane where a mirror is fixed. On the other side, a flat piece of glass was glued to seal the cavity. Notice the attached tube.

**Figure 5:**
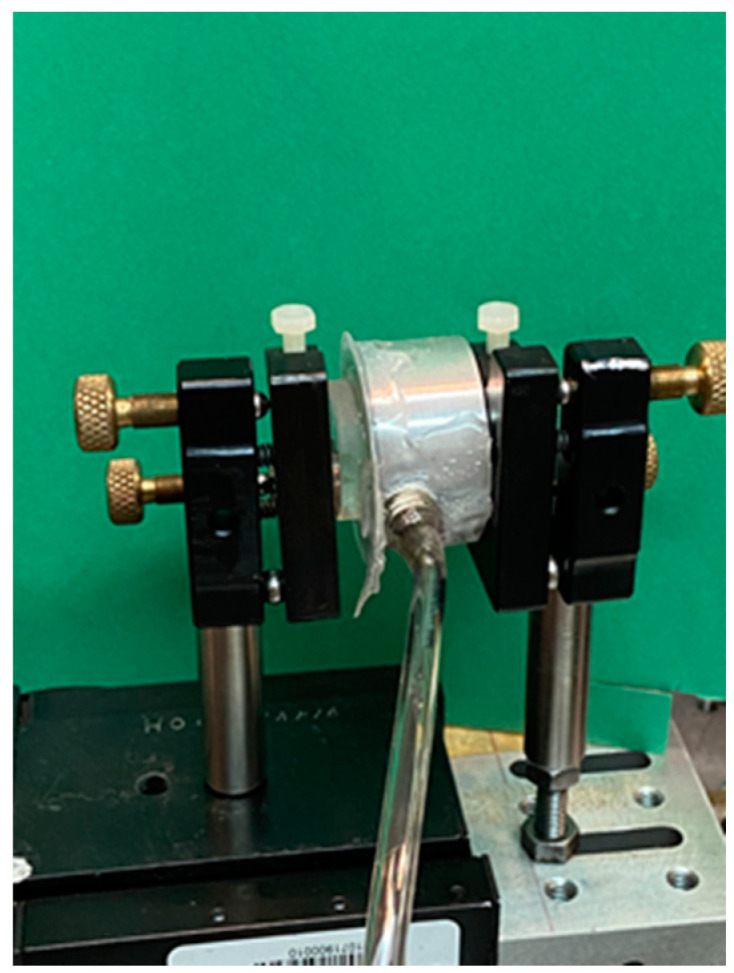
The Fabry-Perot interferometer assembly, showing both mirrors on mechanical mountings.

In the past, the Fabry-Perot system has been used to measure humidity and pressure [21,22]. Very low-pressure values in the order of tens of Pascals have been measured. Here, we have adapted the Fabry-Perot system to obtain the arterial pulse pressure waveform. The configuration is shown in Figure 3. After the light leaves the laser, a microscope (objective 10×) focuses the light, which then travels and reaches the Fabry-Perot mirrors that are 20 cm away. They have an internal reflectance of about 97% at 632.8 nm. On the mirrors, their outer surface has an anti-reflection coating (λ = 632.8 nm). The diameter of the mirrors is 25 mm, the thickness is 5 mm, and the weight is 5 g. They are made from BK-7 glass. The metallic mirror cell used in the experiments is shown in Figure 4. The cell dimensions are 2 cm thick, the front face is 3.5 cm diameter, and the back face is 2.1 cm. The first Fabry-Perot mirror is placed on a mechanical mounting. The second mirror is glued to a silicone membrane, 20 µm thick, and this is attached to the front face of the cell (Figure 4). On the cell rim, a tube (Parker parflex series U 6 mm O.D. × 1 mm wall; 8.6 BAR <125psi> w.p. at 23cc 3803714306) is connected to the acrylic sensor (Figure 5). The distance between the mirrors is 5 mm. A lens placed 5 cm after the Fabry-Perot system focuses the light on an iris with an aperture of 500 µm (diameter). The electronic detector was placed after the iris. The lens has a focal distance of 30 cm. The selected membrane thickness is enough to allow for mirror movements due to the blood pulsing.

Considering that the He-Ne laser wavelength is λ = 632.8 nm for a silicon detector, a Newport silicon PIN detector model 882 was used. The peak responsivity wavelength, λp, is at 850 nm, and at a responsivity of R = 0.45 A/W @ λp, the rise time is 2 μs. Responsivity at this laser wavelength decreases to about 0.30 A/W, with a sensing area of 1 cm^2^. Since the detector has very good responsivity, no amplifier was built; the detector had a direct connection to the digital oscilloscope input in the photovoltaic mode.

### 5.2. The Pressure Sensor

**Figure 6:**
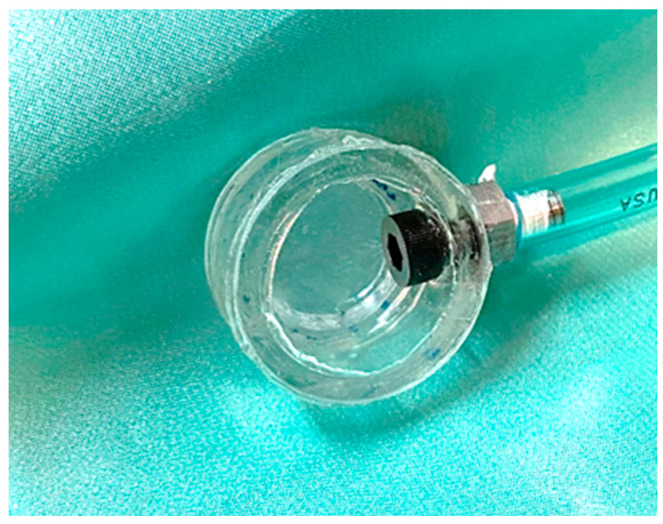
The pressure sensor. An acrylic tube has a thin, flexible membrane that can be put in contact with the wrist artery. On the other end, a flat piece of glass sealed the cavity. A tube was attached to the rim of the tube.

Pressure can be monitored by several means. One way is by using membranes. Here, the suggested sensor consists of a circular acrylic plastic cylinder, 15 mm thick and 31 mm in diameter, which, on its front face, had a glued thin silicone membrane and, on its back face, a glued piece of flat glass was used to seal the cavity. On the rim, there is a connection to a hose (Figure 6). Several membrane thicknesses were used with values of 20 μm, 200 µm, 400 μm, and 1 mm. For the last three values, it was noted that the response time was long; thus, the 20 µm membranes were used.

When the heart pumps blood through the arteries, a blood pulse travels and creates a traveling bump. The sensor has a flat, stretched silicone membrane that is placed in contact with a wrist artery. When the bump passes under the membrane, an air pulse is created that travels through the sensor tube. When the air pulse arrives at the mirror cell, it produces an axial mirror movement that, in turn, gives a radial movement to the interference fringes; this is captured by the electronic sensor.

### 5.3. Testing the Sensor Unit

**Figure 7:**
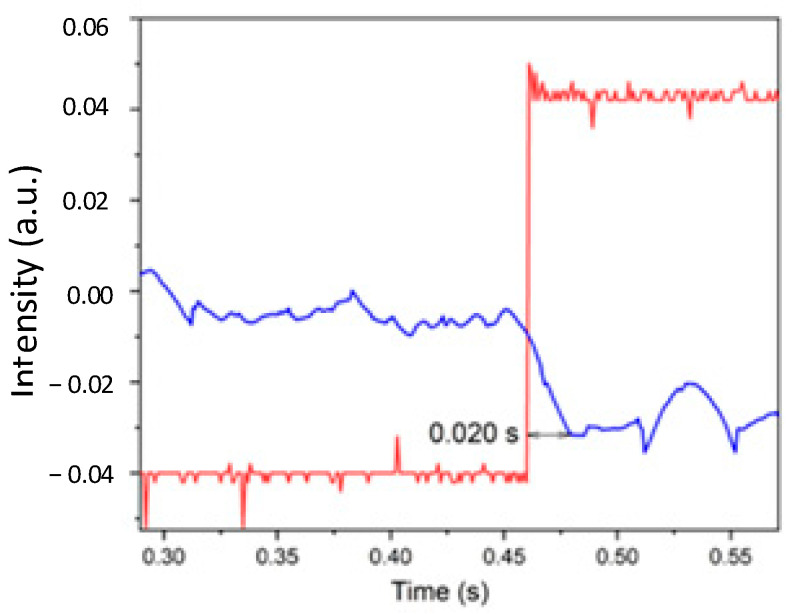
Two waveforms are shown. The square version was given by the frequency generator and the other by the detector that gathered interference pattern movement.

**Figure 8:**
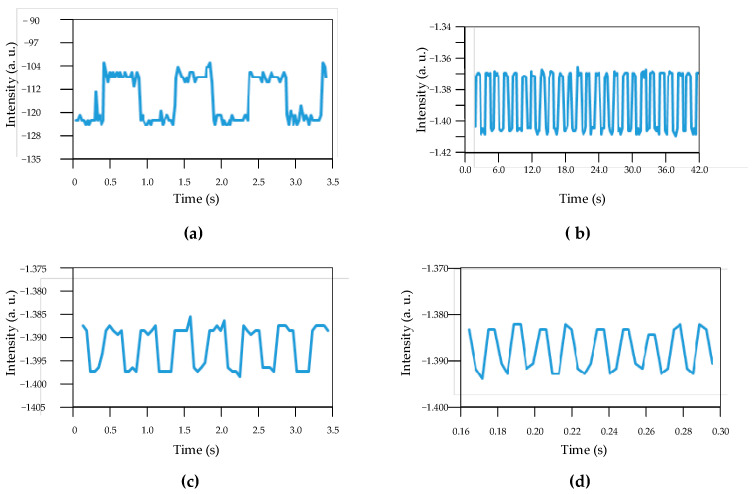
Response of the acrylic sensor to different frequencies. (**a**) 1Hz, (**b**) 6 Hz, (**c**) 45 Hz, and (**d**) 90 Hz.

In order to test the quality of the system frequency response, we connected the sensor membrane to an audio speaker for the purpose of simulating a driving pulse with a known wave shape. We used a square wave test waveform with some selected frequencies. By using an oscilloscope, we observed both the driving signal and the sensor signal output. Four selected frequencies were used: 1 Hz, 6 Hz, 45 Hz, and 90 Hz. Figure 7 shows the obtained data at the oscilloscope. The driving square pulse is in red, and our sensor output pulse is in blue, as shown in Figure 7. The sensor output pulse has a delay of about Δt = 20 milliseconds with respect to the driving pulse. A voltage amplitude of about Δv = 0.02v gives a voltage slew rate close to s = 1 *v*/*s*. In Figure 8, we can observe the response for the driving frequencies of 1 Hz, 6 Hz, 45 Hz, and 90 Hz. It is noted that a good response was recorded for square pulses of 1 Hz and 6 Hz (Figure 8a,b), respectively, since the square shape for the pulses for both figures is still in a square form. For the case in Figure 8d, the wave shape is no longer a good square wave. The slew rate has decreased noticeably. The signal amplitude maintains the same level as in the previous cases; therefore, the amplitude for the base frequency has no noticeable attenuation. Since the wave is now approaching a sinewave, fifth and higher harmonics attenuate. With a first approximation, we can estimate that the system starts losing response at the fifth harmonic (about 450 Hz).

From the appearance of the waveform in Figure 8d, we conclude that the system responds as a low-pass filter with a bandwidth of at least 500 Hz. The waveform shows no ringing; therefore, the system does not have significant resonance. In addition, from the four cases in Figure 8, no significant noise is present, whereas in Figure 7, there is some noise, which we can attribute to being captured by the tubing acting as part of the transduction process. This suggests working on the quality of the tubing for noise insolation. This is a future task that warrants our attention.

### 5.4. Experimental Arteries Pulse Waveform

**Figure 9:**
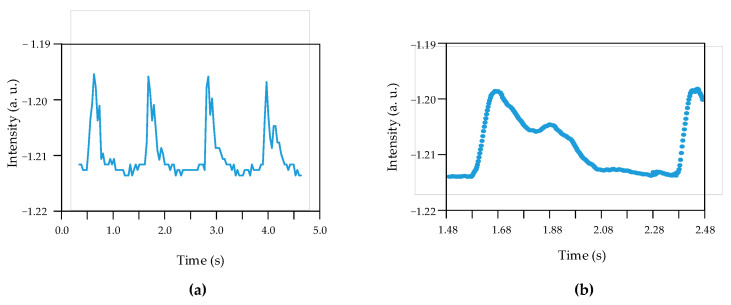
(**a**) Pulse rate of a young person. (**b**) Detailed view of a cycle.

A young female (25-years-old) participated in the sensing tests. An analog-to-digital card was used to record the light variations of the interference pattern onto computer memory. In Figure 9a, a plot of the pulse rate is shown. Figure 9b shows a detailed view.

By showing a relation of the plot (Figure 2), described in Section 4, to the plot in Figure 9b, the first peak is given at the time 1.6747 s, and the second peak is given at 1.8882 s. The difference in time is ΔTDVP = 213 ms. Regarding the augmentation index (Air), which is given by the ratio of the second peak (P2) to the height of the first peak (P1), it is 0.59. These values (213 ms and 0.59) are compatible with the values given for a human being of that age [23].

From the previous data, it is possible to calculate instrument sensitivity. The sensitivity for an electrical pressure meter is defined as s = (ΔV/V_o_)/ΔP, with ΔV being the variation in the voltage for a given pressure variation, ΔP, and V_o_ is the baseline voltage. According to Huang et al. [24], a typical radial pressure is about 50 mmHg, corresponding to 6.67 kPa. The corresponding voltage variation from Figure 9a is ΔV = 0.015; therefore, the sensitivity is 1.85 kPa^−1^.

Regarding the sensitivity of the device shown in [10], the sensitivity is 8.4 kPa^−1^. The device in [8] has a sensitivity of 3.73 kPa^−1^. The device presented in this manuscript is a little lower: 1.85 kPa^−1^.

Through this work, it has been shown that a Fabry-Perot interferometer can be adapted to measure arterial pulse. As part of the characterization process of the Fabry-Perot system, the response to several frequencies and the response time are shown. In Table 1, the characteristics of various electronic pulse meters are shown along with the optical Fabry-Perot characteristics.

As can be seen in Table 1, the Fabry-Perot system has a response time compatible with other electronic sensors. Nevertheless, electronic sensors are at a disadvantage because their method of fabrication is long, cumbersome, requires dedicated electronic semiconductor facilities, personnel, and power sources, can be affected by electronic radiation, and contributes to e-waste. The optical pulse wave device based on a miniature Fabry-Perot fiber optical configuration, which is mentioned in Refs. [14,15] is complicated because it uses elements such as a SLED Exalos EBD5000 driver board, an Ibsen I-MON USB512 spectrometer, an optical fiber circulator, and a computer. Moreover, the fabrication of the miniature Fabry-Perot system is cumbersome.

## 6. Conclusions

The device that is presented here can be adapted to industrial, healthcare, and biomedical applications, to mention but a few. The Fabry-Perot system has some advantages over rigid electronic sensors because it presents electromagnetic immunity, corrosion resistance, electrical isolation, environmental friendliness, chemical resistance, and sensitivity, is economical, takes data noninvasively, shows bio-compatibility, and uses fewer and simpler components in the optical configuration means less trouble for a given application. Its sensitivity can be adapted according to the mirror fabrication method and by changing the thickness of the silicone membranes. Thin membranes are more flexible, thus increasing the sensitivity of the device; on the other hand, thick membranes are more difficult to flex; thus, physical situations with more rough movement could be monitored using thicker membranes. A reticulated three-dimensional structural network is formed inside the silicone films, providing properties such as flexibility and elasticity with shape memory, returning to its original shape when any force is released. The frequency of monitoring can range from less than 1 cycle/s to about 90 cycles/s.

## Figures and Tables

**Table 1:** Comparison between our own and other methods used to measure arterial pulse.

Method or Material	Response Time	Based on Light or Electronics	Fabrication Procedure	Reference
Polypropylene film	~21 ms	Electronics method; capacitive measurements	Complicated sensor fabrication process; use of semiconductor electronic fabrication facilities; health-threatening; e-waste	Wen Cheng IEEE Ref. [7]
MXene (TiC_2_Tx)-coated non-woven fabrics layer; Flexible pressure sensor	~15 ms	Electronics method; increases conductive fiber paths during compression	Long chemical fabrication process to make MXene (TiC_2_Tx)-coated non-woven fabrics layers; use of electronic fabrication facilities; health-threatening; e-waste	Weihao Zheng Micromachines Ref. [8]
Lead zirconate titanet	~0.1 ms	Electronics method; Piezoelectric and semiconductor materials; capacitor-type structure	Use of MOSFET to amplify the signal; use of electronic fabrication facilities; health-threatening; e-waste	Canan Dagdeviren Ref. [9] Gregor Schwartz et al. Ref. [10]
Tonometry	No data	Micro-manometer-tipped probe on carotid; results compared with micro-manometer–tipped catheter, placed in the aorta near the heart	Need for electronic fabrication facilities; the use of a catheter inside the arteries could cause infections	Chen-Huang Chen Ref. [13]
Optical Fabry-Perot with flexible membrane	~20 ms	Light method; optical interferometry	Mirror and thin film fabrication method; detector on wrist.	This work

## Data Availability

Data are contained within the article.
